# The Impact of Surgeon’s Expirience and Volume on Pelvic Reconstructive Surgery Outcome

**DOI:** 10.1007/s00192-026-06598-4

**Published:** 2026-03-26

**Authors:** Gil Levy, Zviya Fridman-Kogan, Oshri Barel

**Affiliations:** https://ror.org/05tkyf982 grid.7489.2 0000 0004 1937 0511 Div. of Pelvic Floor Medicine. Dept. of Obstetrics and Gynecology. Assuta Samson University Hospital, Ben Gurion University of the Negev, Negev, Israel

**Keywords:** Surgeon experience, Surgical volume, Pelvic reconstructive surgery, Learning curve, Vaginal mesh, Surgical outcomes

## Introduction

Surgical outcomes are the primary criterion guiding evidence-based selection of operative techniques across all surgical disciplines, including pelvic reconstructive surgery. Outcomes are most commonly reported as perioperative complications, anatomic success, and patient-reported symptom improvement over short-, mid-, or long-term follow-up [1–4]. However, these outcomes reflect not only the intrinsic effectiveness of a procedure but also the context in which it is performed. Surgical performance is influenced by multiple interacting factors, including patient comorbidities, case complexity, institutional resources, and—critically—the surgeon’s experience and the hospital’s procedural volume [5].

Although randomized controlled trials (RCTs) and comparative cohort studies are regarded as the highest level of evidence for evaluating surgical interventions, they frequently assume that the technical execution of procedures is uniform across surgeons and institutions. In practice, this assumption is rarely valid. Most surgical procedures have a defined learning curve, during which complication rates, operative time, and clinical outcomes evolve as surgeons acquire proficiency [6, 7]. True procedural mastery is achieved only after a threshold number of cases is reached, beyond which outcomes stabilize at a plateau level [8, 9]. Similarly, high-volume institutions may benefit from standardized care pathways, experienced surgical teams, and improved perioperative management, all of which may independently influence outcomes.

Despite this, surgeon experience and institutional volume are often insufficiently reported, inadequately controlled for, or entirely ignored in clinical trials and systematic comparisons of pelvic reconstructive procedures. As a result, it remains unclear to what extent observed differences in outcomes between surgical techniques reflect the procedures themselves rather than differences in surgeon expertise or procedural volume. This represents a critical knowledge gap in urogynecologic surgery, where complex reconstructive procedures are highly operator-dependent and widely adopted on the basis of published comparative effectiveness data. To address this gap, this review was guided by the following question: To what extent do surgeon experience and institutional surgical volume influence perioperative, anatomical, and patient-reported outcomes in pelvic reconstructive surgery, as reported in randomized and comparative clinical trials? The objective of this review was to systematically evaluate the existing randomized and comparative clinical literature—predominantly within urogynecology—to identify and synthesize evidence on how surgeon experience, procedural familiarity, and hospital volume are reported, controlled for, or analyzed, and how these factors impact surgical outcomes when comparing pelvic reconstructive techniques. By explicitly examining these variables, this review aims to clarify whether current evidence allows valid conclusions about the relative effectiveness and safety of pelvic reconstructive procedures independent of provider-related factors. Although the primary focus is urogynecology, the findings have broader relevance to surgical research in general, as they address fundamental methodological issues affecting the interpretation of comparative surgical outcome studies.

## Materials and Methods

This review was not prospectively registered in PROSPERO. Registration was not undertaken because the review was designed as a targeted methodological and conceptual synthesis examining how surgeon experience and institutional volume modify surgical outcomes rather than as a quantitative meta-analysis of a single intervention. The scope evolved iteratively during the scoping phase as learning curves, volume–outcome relationships, training systems, and organizational factors were identified. This approach is consistent with integrative systematic reviews addressing methodological sources of bias rather than pooled treatment effects. This review was guided by the following question: How do surgeon experience, learning curves, and institutional procedural volume influence perioperative and postoperative outcomes in pelvic reconstructive and benign gynecologic surgery, and how are these factors accounted for in comparative clinical studies? A systematic literature search was performed in PubMed/MEDLINE, Scopus, and Web of Science Core Collection in accordance with PRISMA 2020 guidelines. Searches were limited to human studies published in English between January 2000 and March 2025. Reference lists of key articles and reviews were manually screened. The search strategy was based on following Boolean combination across databases: (“surgeon volume” OR “hospital volume” OR “learning curve” OR “CUSUM” OR “experience curve”) AND (“gynecology” OR “hysterectomy” OR “pelvic reconstructive surgery” OR “pelvic organ prolapse” OR “urogynecology”) AND (“outcomes” OR “complications” OR “operative time” OR “training” OR “resident” OR “fellowship”). Eligibility criteria: Studies were included if they evaluated perioperative, postoperative, anatomical, or patient-reported outcomes in relation to surgeon experience, learning curves, or procedural volume in benign gynecologic or urogynecologic surgery. Eligible designs included randomized trials, observational cohorts, registry analyses, and systematic reviews with extractable data. Simulation-only studies, case reports, editorials, and narrative commentaries were excluded. The search identified approximately 1200 records. After deduplication and screening, 132 articles underwent full-text review, of which 35 met all eligibility criteria and were included in the qualitative synthesis. The selection process is summarized in a PRISMA 2020 flow diagram (Fig. 1). Included studies were classified into four predefined domains: (1) high- versus low-volume surgeon or hospital comparisons; (2) learning-curve modeling (including CUSUM); (3) training systems and surgeon preparedness; and (4) ethical and organizational implications of volume–outcome relationships. Extracted data included study design, procedure type, volume or experience metrics, outcome measures, and principal findings. Risk of bias and study quality: Randomized trials were evaluated using the Cochrane Risk of Bias 2 tool. Observational and registry studies were assessed using the Newcastle–Ottawa Scale. Learning-curve and CUSUM studies were evaluated using a structured methodological framework assessing case sequence completeness, outcome definition consistency, appropriateness of statistical modeling, and clarity of plateau definition. Study quality was considered during synthesis but not used as an exclusion criterion. This review was not prospectively registered in PROSPERO, which may increase the risk of selection or reporting bias, although the methodology was predefined and transparently reported. Included studies were heterogeneous in design, outcome definitions, and volume metrics, precluding meta-analysis. Surgeon experience and institutional volume were variably defined, increasing residual confounding. Publication bias cannot be excluded, and most data originate from high-income healthcare systems, limiting generalizability.

**Fig. 1:**
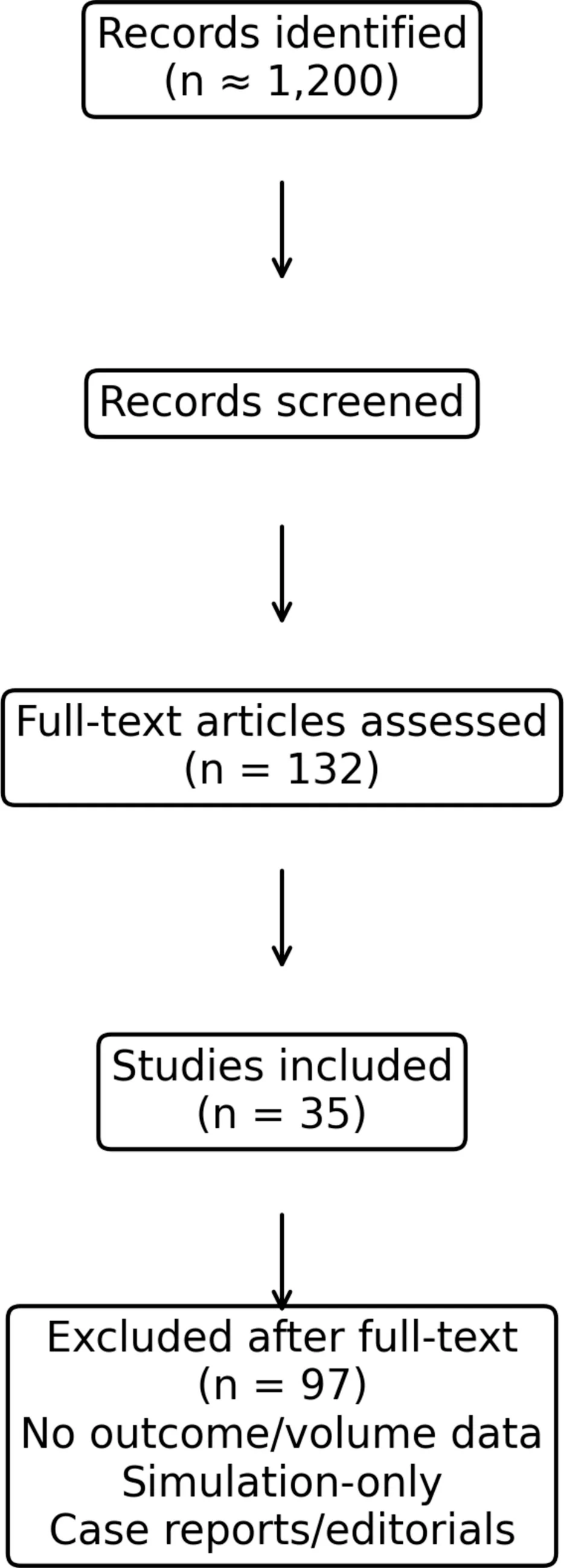
Prisma flow diagram

## Results and Discussion

This systematic review demonstrates that surgeon experience, learning curves, and institutional procedural volume are important, but inconsistently measured, determinants of outcomes in pelvic reconstructive and benign gynecologic surgery. Although most comparative surgical studies focus on techniques, devices, or approaches, only a minority explicitly account for provider-level factors such as training, cumulative case volume, or procedural mastery. This disconnect represents a major methodological gap in the interpretation of surgical outcomes.

Across the included literature, higher surgeon and hospital volume was generally associated with lower complication rates, fewer reoperations, and reduced resource utilization, but the magnitude and consistency of this effect varied substantially by procedure. A 2016 meta-analysis by Mowat et al., including 741,760 gynecologic cases, demonstrated that patients treated by low-volume surgeons had a 40% higher risk of any in-hospital complication, an 80% higher risk of intraoperative complications, and a 50% higher risk of postoperative complications, with even larger differences observed for gynecologic oncology [4]. Although case-mix adjustment was limited, number-needed-to-treat analyses suggested that one in-hospital complication could be prevented for every ten operations performed by a high-volume surgeon. These findings support the conclusion that provider volume is a clinically meaningful modifier of surgical risk.

### Surgical RCT

The study of medicine has taught that the optimal way of providing medical evidence in order to establish a medical treatment was by designing a randomized control trial (RCT). Thus, “Evidence based medicine” has been the foundation for guidelines directing our daily medical practice. Recently, regulatory bodies have become more involved in our medical decisions. Their traditional responsibility, to ensure patient safety, has now been expanded to include treatment efficacy. This change requires a thorough and detailed examination of the tools used to reach these decisions. The double-blind randomized controlled trials have been used successfully to evaluate pharmaceutical therapies. However, the literature supports the conclusion that this method has drawbacks, and it must be modified when evaluating surgical techniques. Urogynecology is considered, at least in part, a surgical discipline. Many surgical techniques were established through a trial-and-error process but in the era of “evidence-based medicine” these techniques require RCTs to establish their safety and efficacy. In the design process of an RCT for a surgical technique we are facing multiple challenges: 1. Should the control arm be another technique or sham surgery? Obviously, there is a major issue obtaining consent from a patient for a study that may include sham surgery—this is probably impractical. 2. Blinding the surgeon is challenging. 3. Ethical issues in the randomization process. The investigator is required to explain to the candidate that the final decision regarding their surgery will be made in the operating room by opening an envelope that determines which technique the surgeon uses regardless of the surgeon’s skills, experience, or preference. To test a surgical technique, the surgeon needs to master the technique through a process we call: the learning curve. Different techniques require different learning curves and these differ between surgeons. Thus, learning curves need to be established for each procedure to be tested, prior to allowing a surgeon to participate in an RCT. Operationalizing learning curves for credentialing and payer policy is beyond the scope of this review. Another factor in the design of an RCT is “Equipoise.” Clinical equipoise is the assumption that there is not one “better” intervention—for either the control or experimental group—during the design of an RCT. A true state of equipoise exists when one has no good basis for a choice between two or more care options. Clinical equipoise has also been called an honest null hypothesis or a state of uncertainty. Similar to clinical equipoise, personal equipoise exists when the surgeon involved in the research study has no preference or is truly uncertain about the overall benefit or harm offered by the treatment to their patient. The ability of the surgeon in an RCT to meet the definition of personal equipoise is challenging.

### The Learning Curve and the Mastery Level

Malcolm Gladwell in his popular book “Outliers” argued that the mastering of any skill requires 10,000 h, or 20 h a week for 10 years. It is safe to say that surgery falls under that requirement. The surgeon’s skill level has a significant impact on the outcome of the procedure. This was demonstrated in multiple disciplines including: cardiovascular surgery, rectal, breast, or pancreatic procedures [10–13]. It should be emphasized that each procedure has its own learning curve. Notwithstanding, the learning curve is not the sole factor to determine the risk for complications. In certain procedures the learning curve might be different than the number of cases required for the risks to reach a stable plateaued complication rate, which should be defined as the mastery level. Thus, even the definition of high- vs low-volume surgeon itself presents a challenge. Master level might not be achieved in all procedures [14]. A 2019 publication in Annals of Surgery by Yu et al. reviewed all the surgical RCTs published in 2013 [12]. Of 200 analyzed studies, 43% were multicenter studies; 85% tested eight alternative interventions; 45–75% included randomization techniques and only 48% addressed the blinding of the assessor. The quality of the surgery was measured objectively, only in 24% of the studies and in 12% by compliance measures. There was no effort to consider the learning curve of the tested surgical techniques. Regarding the surgical skills, only 47% and 30% of studies, respectively, controlled for quality of the surgical intervention: by either limiting the criteria for surgical eligibility or by the surgeon’s experience in years of experience, case load or professional grade. The authors concluded that RCTs in surgery did not address major issues as complexity of the procedures, learning curve evaluation, surgeon skill eligibility, training, previous number of cases, or qualifications [12]. Another factor that influences the learning curve and surgical volume is the residency and subspecialty training. Multiple publications raised the question of the quality of surgical training during residency with a wide variety on the surgical skills achieved at the end of the basic OBGYN training residency [15–18]. There is a consensus that overall the current residency programs syllabus are lacking the tools to evaluate personal surgical skills of the individual resident. A study by Warner, Brown and Bretschneider in 2022 [19], using the American College of Surgeons National Surgical Quality Improvement Program (ACS-NSQIP) compared surgical outcome of pelvic prolapse repair between obstetrician-gynecologists and trained urogynecologist. A significant 30-day postoperative complication rate and reoperation rate reduction was documented in the subspecialty group. No difference was documented in readmission between the groups. Using a multivariant regression model the authors calculated nearly 40% lower odds ratio of any 30 days post operative complication in procedures performed by trained urogynecologists. Regulatory rules play an important factor that is usually ignored in surgical publications. Regulatory reviews have leaned on large datasets, multicenter RCTs/registries, and long-term patient-reported/anatomic outcomes. These choices appropriately prioritize safety and real-world generalizability. Surgeon and center effects are incompletely measured or omitted. Multiple studies show sizable, directionally consistent advantages for high-volume surgeons/centers (e.g., 40–80% relative reductions for several complication classes in pooled benign gynecology, and strong center-volume effects in complex reconstructions) [4, 20, 21]. For specific procedures, thresholds matter: ≥ 13 mesh POP cases/year associated with 41% lower mesh reoperation risk [22]; MUS shows lower reoperation with > 40/year and a plateau around ~ 110 lifetime cases [23, 24]. Learning curves are rarely incorporated. CUSUM analyses document early penalties and subsequent stabilization for vaginal reconstructions, pectopexy, radical hysterectomy, and singlesite laparoscopy [6–9]. RCT audits show little control for skill/learning curve in trial design and conduct [12]: nonuniform definitions of “high volume”: heterogeneous thresholds (percentiles vs absolute counts; annual vs career totals) generate between-study variability and complicate pooling. Case-mix residual confounding: high-volume surgeons may take more complex cases. Without stratification, effects may be biased toward the null (for benign procedures) or away from the null (for complex reconstructions).

To demostrate the extent surgeon factors could change pooled estimates, we analyzed the following usecase: POP treated with mesh vs native tissue: reoperation risk: observed (example composite across studies): mesh 6.0% vs native 4.0% → RR(obs) = 1.50. Surgeon-volume effect from Kelly et al.: surgeons ≥ 90th percentile (≥ 13 mesh POP cases/year) have 41% lower mesh-related reoperation vs lower-volume operators [22]. Assume in a typical dataset: mesh cases are 60% low-volume (LVS) and 40% high-volume (HVS); native tissue cases occur mostly with LVS for POP (e.g., 70% LVS, reflecting practice dispersion). Let baseline mesh reoperation risk if all cases were LVS be 6.0%. Apply the 41% reduction to the HVS stratum:

- Mesh (stratified):
  o LVS 6.0% × 60% = 3.60%
  o HVS (6.0% × 0.59) × 40% = 1.42% 224
  o Mesh total (mix) = 5.02% 225
- Native tissue (no strong surgeon-volume effect reported for failure at 1 year in large series [25]; keep 4.0%): 4.0
- RR (adjusted for volume mix) = 5.02 / 4.00 = 1.26 (down from 1.50).

A realistic surgeon-volume adjustment can shrink the apparent harm signal by ~ 16–25% relative, potentially changing strength/grade of evidence and risk–benefit thresholds. If mesh trials/registries enriched for HVS (e.g., 70–80% HVS)—the scenario expected in credentialed centers—mesh reoperation falls to ~ 4.5–4.2%, and RR vs native tissue approximates 1.12–1.05, often nonsignificant once uncertainty and case-mix are considered.

### Hospital Volume

Table 1 summerizes, procedure-by-procedure synthesis depicting where surgeon/hospital volume (and experience/learning curve) most significantly influence patient outcomes. Volume/experience matters most in the following procedures: strongest, most consistent volume effects: overall gynecology and complex pelvic reconstruction; mid-urethral sling (MUS) reoperation; benign hysterectomy complications and resource use; colorectal endometriosis flaps. Mixed/weak effects: native-tissue anterior/posterior colporrhaphy (no clear advantage of high volume on failure at 1 year in a large Swedish series). Clear learning-curve signals: CUSUM/series show early performance penalties for pectopexy, vaginal prolapse reconstructions, radical hysterectomy, and single-site laparoscopy. Analysis is limited by the heterogeneity of these studies: Definitions of “high volume” vary widely (percentiles, absolute counts, annual vs lifetime cases). Outcome baskets differ (composite complications vs specific adverse events, short- vs long-term reoperations). Case-mix and complexity are often imperfectly adjusted (e.g., HVS may take on tougher cases). Settings span national admin databases, single-center series, and registry analyses. Given these, Table 1 reports heterogeneity qualitatively. Where a single meta-analysis exists (Mowat 2016 [4]) the direction and magnitude are robust, but underlying study variability remains high. Another analysis limitation is the quality grading. The following factors need to be considered in quality grading: Most evidence is observational (administrative databases, registries) → baseline low certainty. Upgrades for large effects, dose–response (clear thresholds), and consistency across systems → many domains rise to moderate. Downgrades for residual confounding (case-mix, surgeon selection), inconsistent thresholds, and indirectness (costs as proxy). In a study published in 2006, Sung and her group analyzed the impact of hospital and surgeons’ volume in gynecology in the USA [20]. The Nationwide Inpatient Sample system was used to extrapolate the data of 2986 hospitals in the entire USA. The analysis included 767,000 women operated on per year between 1998 and 2003. Mortality rate was documented at 0.1%. Complication rate was 20%. Hospitals were divided by case load per year. Surgeons’ volume was divided into three categories: low, medium, and high. Women having surgery at low volume hospitals had almost a threefold higher risk of death. No difference was found in the postoperative complications rate among the hospitals with low, medium, or high surgical volume. The effect of hospital volume on surgical outcome is not fully clear. Some have speculated that high-volume hospitals may have improved processes for postoperative care, better-staffed intensive care or nursing units, greater resources to provide complex perioperative care, and more subspecialty surgeons. The outcome examined was limited to complications and nonroutine discharge of the patients. The analyses on impact of surgeons’ volume in the high-volume hospitals, showed significant outcome differences between the HVS and both the medium- and LVS [20]. The difference between high- and low-volume hospitals is more pronounced in complex procedures. Chi et al. [21] studied the cost and outcome of pelvic reconstructions using the American National Inpatient Sample Database. Pelvic flap reconstruction following 274 gastrointestinal or genitourinary tract resections were found to have 49.8% complications. After regression adjustment, high hospital volume was the strongest independently associated factor for significantly decreased surgical complications down to 34.8%.

**Table 1:** Procedure-specific strength of surgeon and hospital volume–outcome relationships in pelvic reconstructive and benign gynecologic surgery

Procedure	Primary volume metric	Outcome(s) influenced by volume	Strength of volume–outcome relationship	Key evidence	Interpretation
Vaginal mesh prolapse repair	Surgeon annual cases (≥ 13/year; 90th percentile)	Reoperation for mesh-related complications	Strong	Kelly et al. 2016 [22]	High-volume surgeons have 41% lower reoperation risk; quartile-based definitions obscure this effect
Mid-urethral sling (MUS)	Surgeon annual cases (> 40/year); cumulative experience (~ 110 lifetime cases)	Reoperation, postoperative retention, recurrent incontinence	Strong	Kaiser Permanente cohort [23]; Ontario registry [24]	Clear dose–response and learning-curve plateau
Benign hysterectomy	Surgeon and hospital annual volume	Complications, length of stay, costs	Moderate–Strong	Mowat et al. [4]; Sung et al. [20]; national datasets [10, 11, 26–30, 30]	Consistent reduction in morbidity and resource use
Complex pelvic reconstruction (flaps, oncologic reconstruction)	Hospital volume	Overall complications, ICU use, costs	Strong	Chi et al. [21]; Sung et al. [20]	High-volume centers achieve substantially lower complication rates
Native-tissue anterior/posterior colporrhaphy	Surgeon annual POP volume	Operating time, length of stay	Weak (efficiency only)	Nussler et al. 2017 [25]	Volume improves efficiency but not failure or complication rates
Vaginal prolapse repair (non-mesh)	Surgeon cumulative experience; CUSUM plateau	Early complications, technical failure	Moderate (learning curve)	CUSUM analyses [6–9]	Early performance penalties with later stabilization
Pectopexy	Surgeon cumulative case number	Operative time, complications	Moderate (learning curve)	Learning-curve studies [7]	Clear learning-curve effect before plateau
Radical hysterectomy	Surgeon experience and case sequence	Complications, oncologic quality	Moderate–Strong	Yu et al. [12]; CUSUM studies [8]	Skill acquisition critical to valid outcome comparison
Single-site laparoscopy	Surgeon cumulative volume	Conversion and complication rates	Moderate (learning curve)	CUSUM analyses [9]	Early learning-phase risk followed by stabilization

### Colporrhaphy

In 2017 a Swedish group led by Emile Nussler [25] was interested in determining why the failure rate of pelvic organ prolapse (POP) surgery in their country was high. Was it due to the surgical technique or the surgical skills? In Sweden 74% of POP surgeries were performed by surgeons with once a month or less experience with the procedure. The authors analyzed 14,676 anterior and posterior colporrhaphy procedures. The definition of surgical volume per surgeon included all POP surgeries performed by a specific surgeon and the experience was defined as volume per year. According to collected surgeons’ reports and patients’ reports, the results showed that experienced surgeons had shorter operating room (OR) time and patients had shorter hospital stay. However, no difference was found in intra-surgical or postop complications, patients’ satisfaction, or failure rate at 1-year. Even the assistant’s skill level did not change the surgical outcome. The final conclusion was that the high failure rate of anterior and posterior colporrhaphy was not related to the LVS. Rather, the authors suggested the results warrant changes in the procedure itself.

### Vaginal Mesh Procedures

In 2016, a Canadian group led by Kelly published their analysis on the impact of surgeons’ experience in vaginal mesh procedures [22]. The authors analyzed 5448 procedures performed in Ontario between 2002 and 2013, with an average follow-up of 5.4 years. The results showed a total of 4% complications rate at an average of 1.17 years. Surgeons’ volume was calculated by quartiles, meaning HVS had above 75th percentile of vaginal mesh cases in a given year—at least five procedures per year. The HVS and LVS groups had 3.9% vs 4.2% repeat surgery due to complications, respectively (not statistically significant). However, when the definition of HVS changed to the 90th percentile—meaning at least 13 cases per year—the patients operated on by HVS had a 41% reduced risk of mesh complications requiring surgical intervention [22]. This is an example of the challenge in defining “learning curve” and “surgeon’s skill” specifically in complex surgical procedures. Another study on the impact of surgical skills on the outcome analyzed the impact of surgical volume on mid-urethral sling (MUS) procedures in the Kaiser health system, which includes 4.5 million members [23]. Currently, in the USA there are 225,000 MUS procedures per year, with a reoperation rate of 5.5%; 3.9% for recurrent UI and up to 3.4% for tape removal at 5 years follow-up. High volume surgeons were defined as performing > 40 cases per year. Patients operated on by HVS compared to LVS had a lower rate of all-cause reoperation (3.6% vs. 4.2%, respectively *p* = 0.04). This is a relative risk of 0.86 and absolute risk reduction of 0.6% of reoperation by HVS. Moreover, patients who underwent surgery by HVS: were 25% less likely to have postoperative stress urinary incontinence that led to reoperation; and were less likely to need prolonged postoperative catheterization [23]. These results are consistent with another Canadian study showing 37% increased risk of reoperation if the sling operation was performed by LVS, defined as performing < 15 cases per year [24]. Overall reoperation was significantly lower in the HVS group, but the mesh removal rate was not found to be significantly different between the groups. The authors tried to analyze the learning curve for MUS. They reached the conclusion that the complication rate plateaued at an average of 110 cases per year [24]. The impact of using data from studies that ignore the surgeon’s experience on everyday practice is substantial. The most recent example of published regulatory decisions which ignored these factors was the dramatic FDA decision to remove all mesh products from the US market [31]. The FDA decided to address both safety and efficacy. In the case of vaginal mesh, a composite outcome was created to include anatomical and subjective outcome. The subjective outcome was based on question number 3 in the PFDI quality of life questionnaire. Length of follow-up was set for 3 years. The published 522 study comparing the Uphold vaginal mesh to NTR ignored the impact of surgeon’s experience that might have changed the outcome. The NTR group was recruited from the AUGS registry which is voluntary—meaning that there is no accurate information regarding surgeons’ volume and experience. These decisions make a major impact on the practice of medicine on a daily basis.

## Conclusions

We have presented in our review the evidence that surgical RCT in gynecology, and specifically urogynecology, lacks critical components, including establishing a learning curve prior to RCT, addressing surgeon’s equipoise, addressing surgeon’s skills and volume that can impact the outcome and addressing hospital volume, as a factor that can impact surgical outcome. The conclusion we’ve reached is that the double blind RCT used in the pharmaceutical world does not provide a reliable methodology in evaluating surgical techniques in urogynecology. We need to encourage the inclusion of factors as the impact of the learning curve and surgeon’s experience on the outcome, in future meta-analysis, registries, and multi-center studies. A modified research design should replace the way we conduct surgical research to establish safety and efficacy of a surgical technique. This design should take into consideration the impact of the surgeon’s skill, volume, and experience on the final assessment of surgical and complications outcomes. Impact of volume and learning curve by procedure can be summarized as follows: MUS and mesh POP: consistent, clinically meaningful reductions in reoperation/complications with higher surgeon/center volume; threshold definitions matter [15, 30–33]. Benign hysterectomy: multiple datasets show lower complications and costs with higher surgeon/hospital volume [10, 11, 26–28, 30, 34]. Native-tissue colporrhaphy: volume improves efficiency (OR time/LOS) but not 1-year failure/complications in a large national cohort [25]—technique quality may dominate. Complex pelvic reconstructions/flaps: hospital volume exerts strong effects on complications and costs [21]. Learning curves: early performance penalties are demonstrable and measurable with CUSUM across several urogynecologic procedures; LC-aware design is essential for fair comparisons [6–9]. Future studies should consider the following factors: Define volume a priori by absolute annual counts and career cases for each procedure (e.g., MUS: < 15/year = higher reoperation risk; centers > 40/year and surgeons with ~ 100–120 lifetime cases show plateaus) [24, 25]. Credentialing and trial eligibility: require documented LC plateau (CUSUM or equivalent) before randomization for technique-comparative RCTs [6–9, 12]. Registry/RCT reporting: always include surgeon volume band, center volume, team composition (subspecialty vs generalist), and case complexity scores [4, 10, 19–28, 34]. Policy: for complex reconstructions (flaps, mesh, oncologic pelvic), consolidate care in high-volume centers to reduce complications and cost variance [21–24]. To incorporate surgeon factors prospectively in future research warrents considering the following factors: 1. Pre-specify volume strata (e.g., < 15, 15–39, ≥ 40 per year; plus lifetime bands) and require reporting by stratum for all device/technique outcomes. 2. Eligibility/credentialing for trials: minimum career cases and CUSUM-verified plateau for each participating surgeon; publish the CUSUM curves [6–9, 12]. 3. Analyze by center volume (tertiles/quartiles) and include team composition (urogynecology subspecialty vs generalist) [19, 21–23, 25]. 4. Regulatory endpoints: Present dual primary analyses—unadjusted (population representative) and credentialed-practice analyses (HVS/HVC strata only). 5. Policy framing: For procedures with strong center-volume effects (flaps, complex reconstructions), prefer regionalization and credentialing over device withdrawal when safety signals attenuate after 374 adjustment. If we agree that surgical experience and volume have an impact on the procedure outcome, then it warrants these to be included in the design of future surgical RCTs used for regulatory purposes.
